# Ship Classification in High-Resolution SAR Images via Transfer Learning with Small Training Dataset

**DOI:** 10.3390/s19010063

**Published:** 2018-12-24

**Authors:** Changchong Lu, Weihai Li

**Affiliations:** Department of Electronic Engineering and Information Science, University of Science and Technology of China, Hefei 230027, China; ll964183@mail.ustc.edu.cn

**Keywords:** synthetic aperture radar (SAR), convolutional neural networks (CNNs), deep learning (DL), ship classification

## Abstract

Synthetic aperture radar (SAR) as an all-weather method of the remote sensing, now it has been an important tool in oceanographic observations, object tracking, etc. Due to advances in neural networks (NN), researchers started to study SAR ship classification problems with deep learning (DL) in recent years. However, the limited labeled SAR ship data become a bottleneck to train a neural network. In this paper, convolutional neural networks (CNNs) are applied to ship classification by using SAR images with the small datasets. To solve the problem of over-fitting which often appeared in training small dataset, we proposed a new method of data augmentation and combined it with transfer learning. Based on experiments and tests, the performance is evaluated. The results show that the types of the ships can be classified in high accuracies and reveal the effectiveness of our proposed method.

## 1. Introduction

Synthetic aperture radar (SAR) is an active Earth observation system that can be installed on planes, satellites, spacecraft, etc. It can perform observations on the ground all day and in all weather conditions. Now we can get more high-resolution SAR images by recent development of SAR satellites, e.g., RADARSAT-2, TerraSAR-X [1,2], etc. By using these images, lots of applications can be implemented. Pieralace et at al. [3] presents a new simple and very effective filtering technique, which is able to process full-resolution SAR images. Gambardella at al. [4] presents a methodological approach for a fast and repeatable monitoring, which can be applied to higher resolution data. Ship classification is an important application of SAR images.

Researchers usually used traditional classification methods in ship classification, including image processing, feature extraction and selection, and classification. Feature extraction is a key step in ship classification. Researchers widely used geometric features, scattering features in feature extraction [5]. For geometric features, it contains ship area, ship rectangularity, moment of inertia, fractal dimension, spindle direction angle and ratio of length to width [6], etc. For scattering features, it contains superstructure scattering features [7], three-dimensional scattering feature [8], radar-cross-section (RCS) [9], and symmetric scattering characterization (SSCM) [10], etc. As for classifiers, artificial neural networks (ANNs) [11] can establish a general classification scheme by training, which makes it widely used in ship classification. Support vector machines (SVM) [12] is also a popular model. Researchers also proposed some methods to get high-classification accuracy [13,14,15], these methods have high requirements for on features and classifiers, so these methods could not applied in other datasets. With the development in neural networks, researchers now focus on processing SAR images with deep neural networks (DNNs) [16,17,18].

The deep neural network (DNN) is artificial neural network (ANNs)’s promotion, which includes lots of hidden layers between the input and output layers [19,20]. DNN can give good expression of an object by its deep architectures and performs well in modeling complex nonlinear relationships [21]. DNN have many popular models, such as recurrent neural networks (RNNs) [22] and convolutional deep neural networks (CNNs). Nowadays, CNNs [23] are playing an important role in detection and recognition. One of the most remarkable results was its application in the ImageNet data set. The ImageNet dataset includes over 15 million images with 22,000 different categories. By using a model called Alexnet [24], the researchers achieved a remarkable result, which have reduced the error rates of 8.2% in top-one error rates and 8.7% in top-five error rates than the previous work [24,25]. Furthermore, CNNs have achieved many impressive results in computer the vision area, such as handwritten digits recognition [26], traffic sign recognition [27], and face recognition [28]. 

Deep learning applications in SAR images have been gradually used. Zhang et al. [5] showed an application in ship classification with transfer learning and fine-tuning. Kang et al. [16] used deep neural networks for SAR ship detection and get good results. Chen et al. [29] presented a method for classification of 10 categories of SAR images, and showed application in ship target recognition. By using deep learning methods, SAR images avoid complicated feature extraction, which completed by deep networks. It can obviously improve the performance of classifiers. However, there are still many problems. Compared with computer vision, SAR image interpretation has the same purpose—extracting useful information from images—but the processed SAR image is significantly different from visible light image, mainly reflected in the band, imaging principle, projection direction, angle of view, etc. Furthermore, the small dataset may be a problem, too. Therefore, when we use the method in SAR images, we need to fully consider these problems.

As with ship classification, many issues may arise when training CNNs. One common issue is over-fitting. Over-fitting can be explained as the neural network models the training data too well and perform bad in data which is different from training data. If a model learns the most of detail and noise of training data, it cannot get good performance in new data, then the over-fitting happens. Some useless information such as noise and random fluctuations have been learned while training as parts of the models. Then the models cannot have good generalize ability in new data. CNNs are prone to over-fitting when model have rare dependencies in the training data. To solve this problem, CNNs often require tens of thousands of examples to train adequately. However, in many cases, we cannot get enough training data in SAR applications, which may cause severe over-fitting. A popular way to solve this problem is data augmentation, such as flipping, brightening, and contrast [30]. Another way to solve this problem is transfer learning, which often been applied in natural images [31].

The application which using deep neural networks in SAR ship images worth paying attention in improving the performance in SAR ship detection and classification. When dealing with SAR images, we should take both the data processed method and CNN training method into consideration. To get good performance, we should retain the important information of the images and get enough images when we make data augmentation and use some good models for training to reduce the amount requirement of training set. In this paper, we proposed a new method for SAR ship classification. First, we proposed a new data augmentation method which can keep important information while increasing the amount of data and achieve the requirements of the dataset. Then, by coupling transfer learning with the processed data, we can get good performance in classification. When dealing with a small training dataset, the method can successfully enlarge the training datasets. With the enlarged datasets coupled with transfer learning, CNNs can avoid the over-fitting issue and achieve excellent classification accuracy. Comparison experiments demonstrated the good performance of our method.

The remainder of this paper is organized as follows. Section 2 presents an introduction of the theory for CNNs, transfer learning and components of our method. The details of the experiments are described in Section 3, we also present our discussion in this section. Finally, Section 4 offers the conclusion.

## 2. Materials and Methods

### 2.1. CNN Models

A CNN is composed of an input layer, an output layer, and multiple hidden layers. The hidden layers can be convolutional layers, pooling layers, activation layers, and fully connected layers [32,33]. The first multilayer CNN is a simple convolutional network consisting of seven levels called LeNet-5 which was proposed by LeCun et al. in 1998 [23]. Authors used it in classifying digits, and it was applied by several banks to recognize hand-written numbers. The pixels of images were 32 × 32. With the large developments in GPU, the layers of CNNs had become much deeper. In recent years, many new models had been proposed, such as Alexnet [24], Resnet [34], VGG16 [22], etc. In this paper, we mainly use two different models for ship classification in order to do comparative test. For further study, we also compared our method with other popular models.

● Traditional CNN models

**Figure 1:**
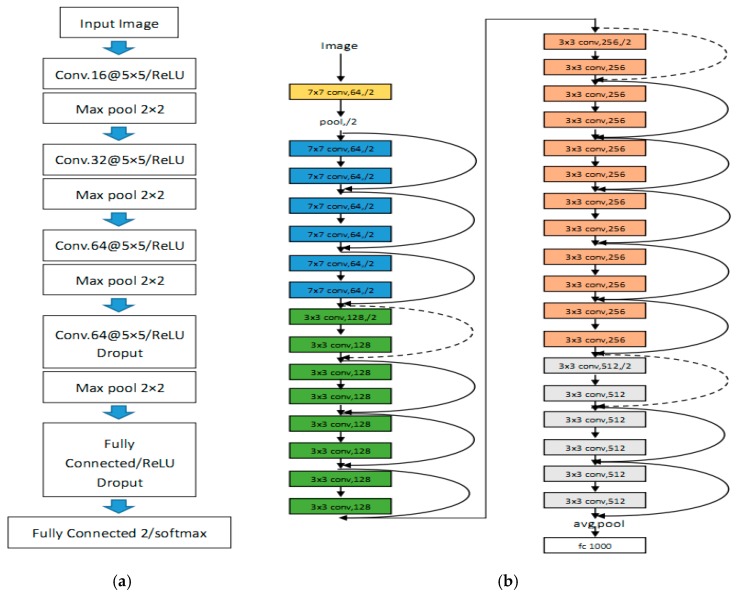
Learning Models. (**a**) Traditional CNN models. (**b**) Resnet-34 models.

The first model we used is traditional CNN models, as shown in Figure 1a. These models consist of four convolution layers, four max pooling layers and two fully connected layers. We used Leaky ReLU as our activation function. To avoid the problem of over-fitting, dropout has been used [35].

● Resnet models

**Figure 2:**
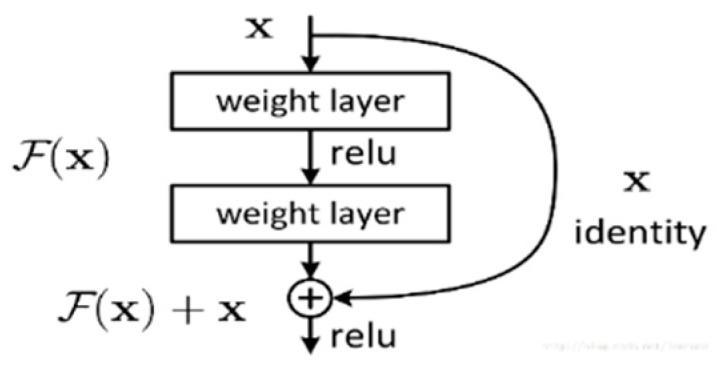
Shortcut connection.

Another model we used is transfer learning the Resnet-50 [34] model. Resnet models used a connection method named shortcut connection, as shown in Figure 2. The models are stacked by multiple blocks. By using this structure, network can obviously improve its performance.

Figure 1b presents Resnet-34 models. Table 1 presents Resnet models with different layers.

● Other popular models 

**Figure 3:**
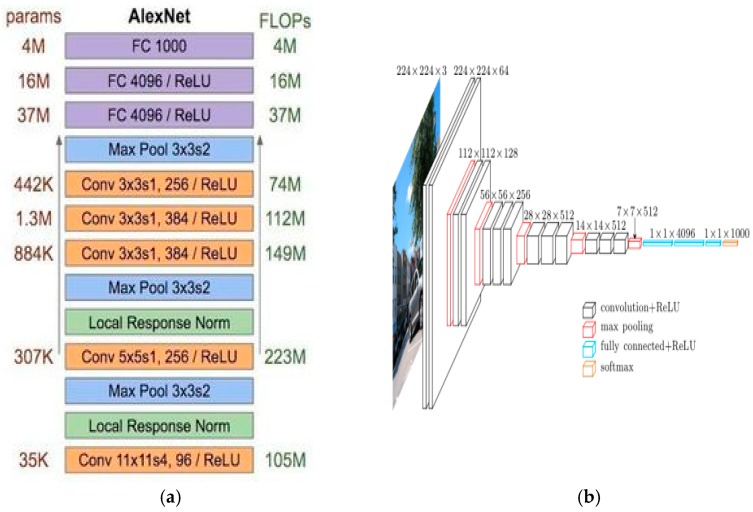
Other popular models. (**a**) Alexnet model. (**b**) VGG-16 model.

In this paper, we also used some popular models to do transfer learning, such as Alexnet, Vgg-16 net, etc. These models are shown in Figure 3.

### 2.2. Learning of CNNs

#### 2.2.1. Environment

The networks are implemented in Pytorch 0.3.0. All layers were designed to match the size of images. The input has a dimension of 224 × 224 = 50176. The output was implemented using the softmax operation and consists of three classes.

#### 2.2.2. Stochastic Gradient Descent with Momentum

Gradient descent is a commonly used optimization algorithm for neural network which can solve many simple problems. However, when the training dataset is very large, we can find that using simple gradient descent method may consume much computing resources, and the convergence process will be slow. At the same time, since all the training data are considered for each calculation in gradient descent method, it may cause over-fitting. To solve this problem, SGD have been proposed.

In stochastic gradient descent (SGD), each training example $x^{\left(i\right)}$ and label $y^{\left(i\right)}$ with be updated as

(1) $$\theta =\theta -\alpha \cdot \nabla_{\theta }J\left(\theta ;x^{\left(i\right)};y^{\left(i\right)}\right)$$

During training, the network only calculates the loss of one sample per iteration, then gradually traverse all samples and complete an epoch calculation. By using this method, although it may produce large fluctuations in a simple, but the result may finally converge successfully. The amount of calculation is greatly reduced, so the speed can also be improved.

Momentum [36] is a commonly used acceleration technique in gradient descent method. Stochastic gradient descent with momentum can be expressed as

(2) $$v=\beta \times v-\alpha \cdot \nabla_{\theta }J\left(\theta ;x^{\left(i\right)};y^{\left(i\right)}\right)$$

(3) θ←θ+v

β is momentum coefficient. It can be understood as, if the last momentum (i.e., v) is the same as the negative gradient direction of this time, then the magnitude of this decline will increase. By using momentum, we can accelerate the convergence process [29]. 

#### 2.2.3. Learning Rate

When deal with CNNs training, learning rate is an important parameter. Usually, we expected to get the result as soon as possible, so the learning rate we used will be large. However, in common situations, by using large learning rate may cause concussion, and the result may not behave as expected. The commonly used method is reducing the learning rate during training. The initial learning rate often takes as 0.01 or 0.001, which is set to decrease the loss function quickly. Then users should adjust the learning rate by several epochs or iterations according to the accuracy during training [29]. The learning rate often been reduced by a factor of 0.1 or 0.5.

### 2.3. Proposed Method with Transfer Learning

#### 2.3.1. Our Method

As mentioned in Section 1, CNNs often require a large amount of data to train adequately. However, as with the SAR dataset, we do not have enough images for training. Therefore, we must do something to avoid the problem of over-fitting.

**Figure 4:**
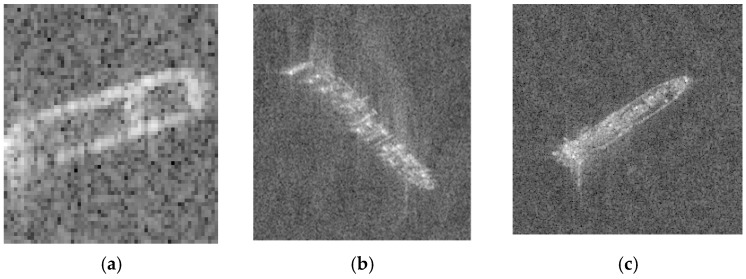
Illustrates the different target classes extracted from SAR images. (**a**) Bulk Carrier with four times magnification. (**b**) Container Ship. (**c**) Oil Tanker.

**Figure 5:**
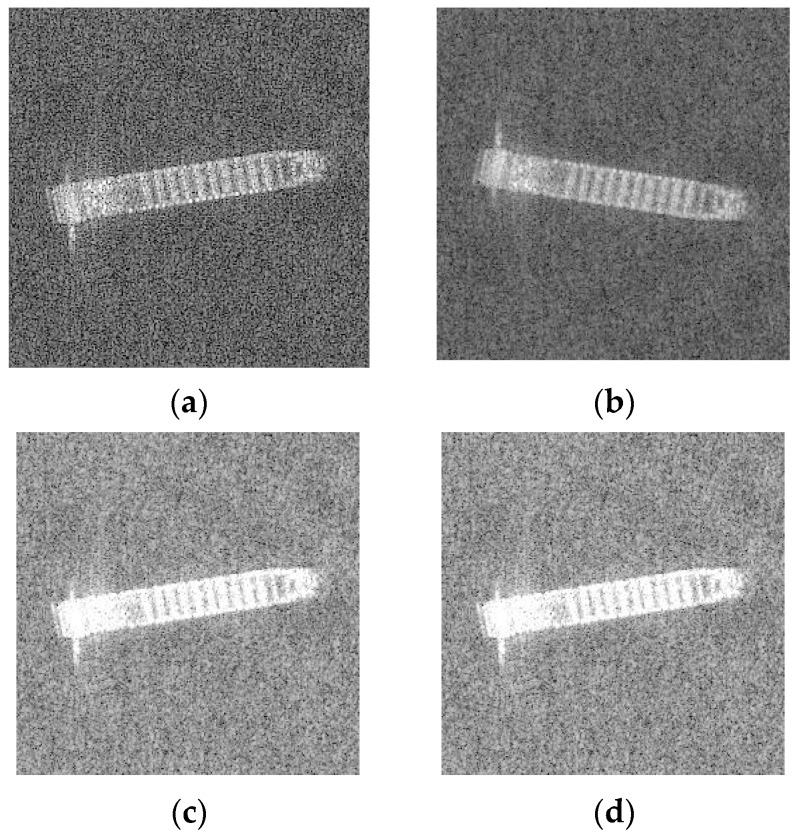
Image and image with flipping, brightening, and sharpness. (**a**) An original image. (**b**) Image with flipping. (**c**) Image with brightening. (**d**) Image with sharpness.

One effective way is data augmentation. There are many methods to achieve our requirement, including adding noise, changing colors, flipping, etc. Figure 4 illustrates the original SAR ship images and Figure 5 shows some common ways of data augmentation. 

One of the most popular methods is random crop. It can significantly increase the amount of data. Recently, researchers usually use some fixed frame to do random crop. For example, using a frame of 224 × 224 to do random crop in a picture of 256 × 256. It not only increases the data, but it also retains the most information of the data.

**Figure 6:**
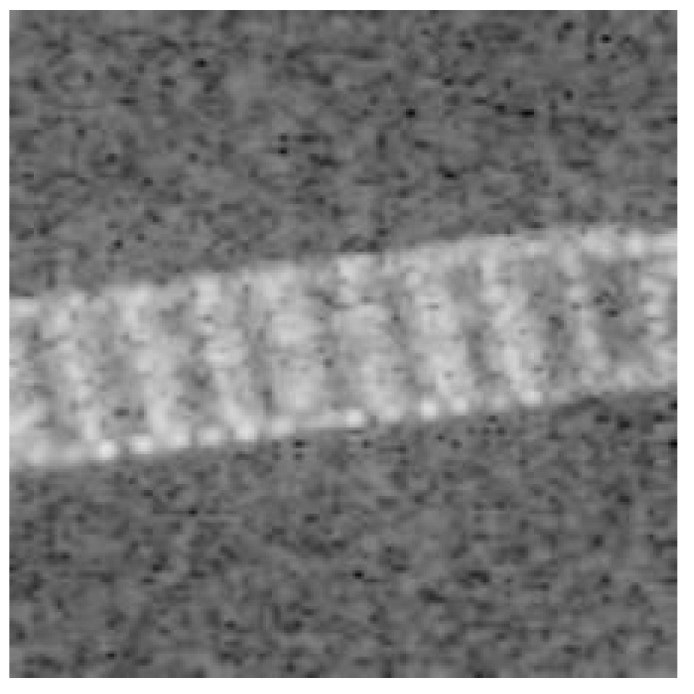
Image with random crop.

However, not all traditional ways of data processing can be helpful when dealing with SAR images. The operation of data processing may loss original information and amplify noise information. Sometimes, random crop may lose some important information. When the main information of images diverges of the center, using random crop cannot perform well, as shown in Figure 6, the bow and the stern of the ship have been cut. In many traditional cases, such as image classification with a cats and dogs dataset, they usually have tens of thousands images for training and testing. When dealing with these problems, though using random crop may lose some information, but they could still keep the main information of images. This information is sufficient for further work. When concerned with SAR images, this method cannot get good performance as expected.

**Figure 7:**
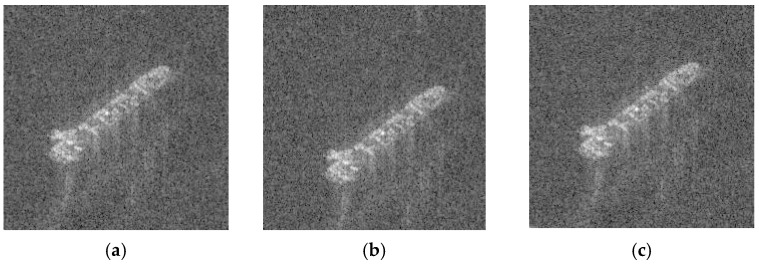
Images with random crop. (**a**–**c**) are three examples of images with random crop.

If we control our crop frame to make sure that the main parts of the pictures haven’t been cut, as shown in Figure 7, it should be noticed that these pictures can be seen as one image and its translations. This method may have good performance in some simple models, which can extract most of the features of the images by training with that data. However, when concerned with deep neural networks, such as Resnet-50, the repeated images cannot give more contributions for training. Thus the performance cannot have more improvement.

**Figure 8:**
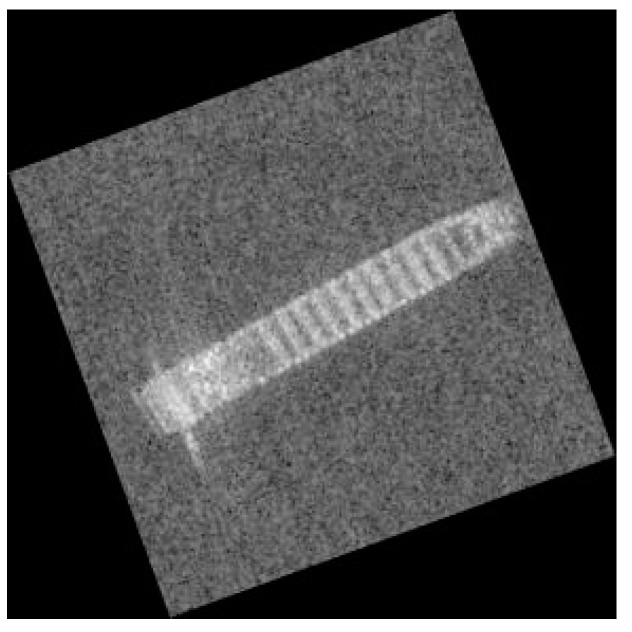
Image rotation of 20 degrees.

Rotating is also a popular way for data augmentation, it can keep main information of images. However, people generally rotate images in an integer multiple of 90 degrees: 90, 180, 270. If we rotate the image by a random number of degrees, some problems may appear. As shown in Figure 8, when we rotate image 20 degrees, the black area may introduce a lot of disturbance into CNN training.

**Figure 9:**
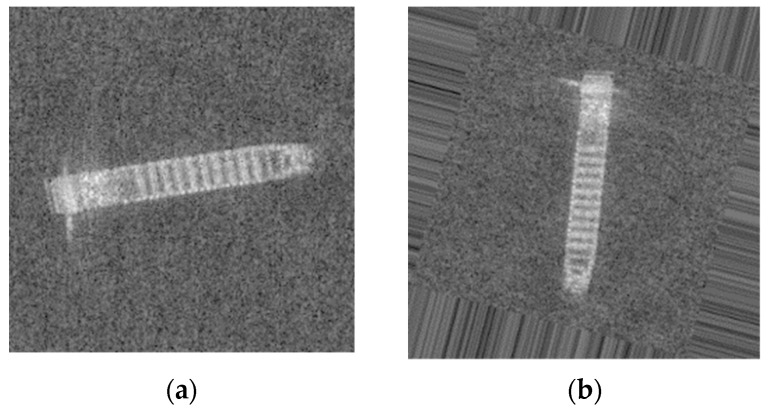
Rotate image. (**a**) Original image. (**b**) Image rotated 256 degrees.

To solve this problem, we expansion the images in their edges with pixel pads to remove the black area. The initial image and the image with rotating have been shown in Figure 9.

By using the proposed method, we not only increase the number of images, but we can also retain the important information of the images. Some noise is still in the background, but concerning SAR images, that noise of the sea surface can be accepted. Because of the characteristics of SAR images, the angle of pictures may cause a lot of difference in CNN training than simple transposition. The data augmentation of all images allows the network to produce better feature representations.

#### 2.3.2. Transfer Learning and Fine-Tuning

**Figure 10:**
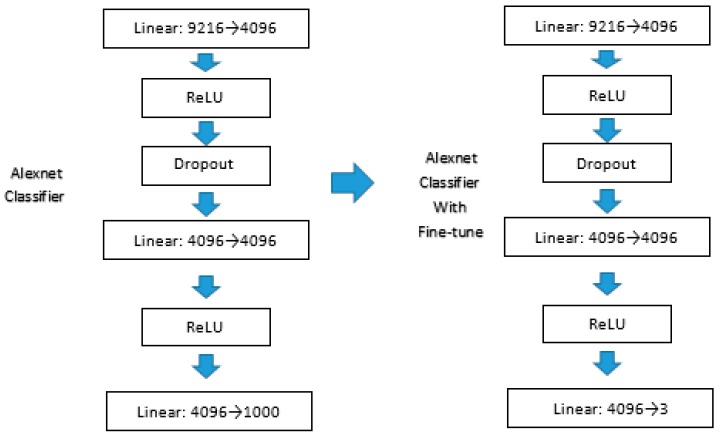
Alexnet with fine-tuning.

Transfer learning is a learning technique that apply the known knowledge from one problem to other related problem [31]. Transfer learning and fine-tune can successfully train a deep model with small datasets. In this paper, we used some pre-trained models for transfer learning, such as Resnet-50, Vgg-16, etc. Depending on structure of different models, we also do some fine-tuning in their layers. The low-level neural layers learned by deep learning models are useful for extracting features such as corners, edges. So, we make the assumption that the lower level neural layers share common features. The methods take the weights of the models in the low-level features as the inputs instead of random weights. Here, we only need to change their fully connected layers and classifiers. For example, the Alexnet model has three linear layers, one dropout layer, and two ReLU layers in its classifier, and the number of its output types are 1000. In our dataset, we need three types for output. So we changed its last layer with output of 3, as shown in Figure 10. The classifier and the fully connected layers should be changed as required.

## 3. Results and Discussions

In this section, experiments are carried out to evaluate the performance of the proposed method. Besides, the comparison with other methods indicates the outperformance of the proposed method. We will discuss the results after our experiments.

### 3.1. Datasets

In this part, we will present our datasets. The SAR ship data set is derived from six full-size SAR images from TerraSAR-X stripmap mode, with a resolution of 2 × 1.5 m in the range and azimuth directions. Slices of SAR ship image are obtained by target detection. And with the aid of the real ground information provided by AIS, all vessels are manually annotated by interpretation experts. The data set includes a total of three types of ships: Bulk Carrier (B), Container Ship (C), and Oil Tanker (OT). A total of 250 stripmap images were used, which were composed of 150 Bulk Carrier images, 50 Container Ship images, and 50 Oil Tanker images. The data size of Bulk Carrier is 64 × 64, and the data size of Container Ship and Oil Tanker is 256 × 256. The chips of ships were shown in Figure 4.

These ship chips are split into training, validation dataset, with percentages of 70% and 30%, respectively. 

### 3.2. Hyperparameters

In this paper, the momentum we set is 0.9. The whole network is trained purely supervised using SGD with a minibatch size [37] of 64 examples, combined with a weight decay parameter of 0.00000001. We rotated images in every 3 degrees for data augmentation. In this paper, the learning rate is initially 0.001 and is reduced by a factor of 0.1 after 100 epochs.

### 3.3. Experimental Data

**Figure 11:**
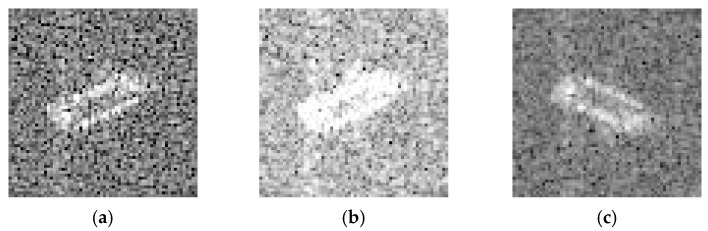

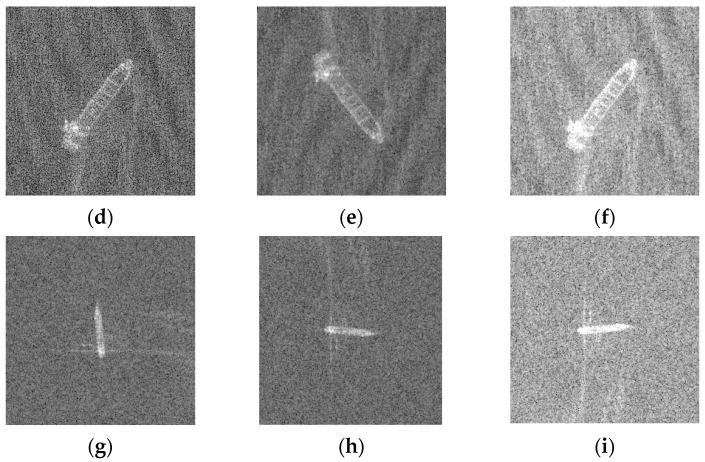
Images with traditional ways. (**a**–**c**) Bulk Carrier images with processing. (**d**,**e**) Container Ship images with processing. (**g**–**i**) Oil Tanker images with processing.

By data augmentation, we have three different datasets, named D1, D2, D3. The D1 classification data set is processed with traditional ways of flipping, brightening, sharpness, etc. The operation we did was shown in Table 2. Therefore, we can expand the dataset seven times. The data are divided into two sets: the training data set Dtrain1 (with 1440 samples) and the validation data set Dval1 (with 560 samples). Samples are shown in Figure 11.

**Figure 12:**
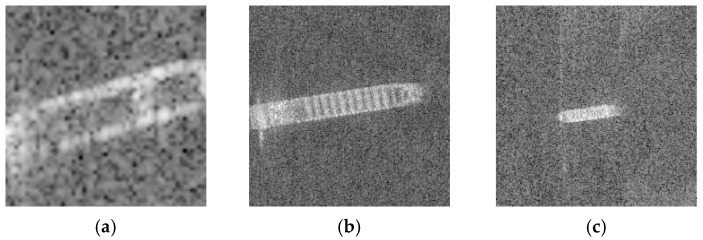
Images with random crop. (**a**) Bulk Carrier image with random crop. (**b**) Container ship image with random crop. (**c**) Oil Tanker image with random crop.

The D2 classification data set is processed with random crop, divided into two sets: the training data set Dtrain2 (with 14270 samples) and the validation data set Dval2 (with 8400 samples), as shown in Figure 12.

**Figure 13:**
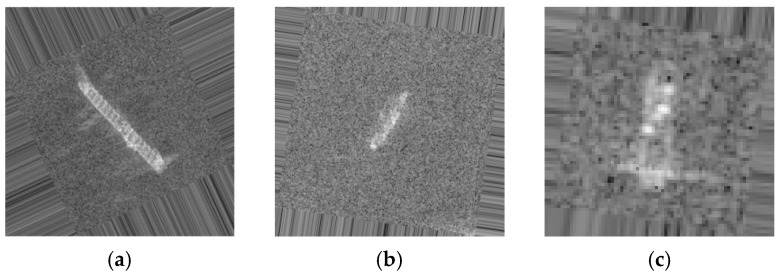
Images with rotate. (**a**) Container Ship image with rotate. (**b**) Oil Tanker image with rotate. (**c**) Bulk Carrier image with rotate.

The D3 classification data set is processed with proposed method, divided into two sets: the training data set Dtrain3 (with 13,823 samples) and the validation data set Dval3 (with 8127 samples), as shown in Figure 13.

These three datasets were trained with models as shown in Section 2.1. The image chips of the dataset are listed in Table 3. To confirm the correctness of our methods, we also use the original image in validation dataset as our test dataset. The test datasets are listed in Table 4.

All of the experiments have been done several times, and we listed the average results of the experiments.

### 3.4. First Experiment: Datasets Using Traditional CNN model

In the first experiment, we used the traditional model, as shown in Figure 1a, deal with D1, D2, and D3 datasets. 

The results of the first experiment are summarized in Table 5, where accuracies achieved by the traditional CNN models on our validation data set are listed for the considered three classes of maritime targets. 

From Table 5, we can see that the traditional CNN models have low accuracy in the D2 and D3 datasets, but perform better with D1 dataset. This is expected, as the traditional CNN model we used have only 10 layers, which is a simple neural network. The D1 dataset has a total of 2000 images for training, so the simple network can produce features pretty well, while when consider with D2 and D3 dataset, the simple network cannot give good expression. Therefore, they had lower accuracy. Furthermore, when using traditional CNN models, it may take more than 1000 epochs when the results become steady.

### 3.5. Second Experiment: Datasets Using Resnet-50 Model

In the second experiment, we used the Resnet-50 models deal with D1, D2, and D3 datasets. The models was shown in Figure 1b.

The results of the second experiment are summarized in Table 6.

As shown in Table 6, these results show that the deep networks with transfer learning have better performance than the traditional CNN models when dealing with the three datasets. We can achieve at least 3% higher accuracies for classification when compared with dataset using traditional CNN model. With deep networks, the images with data augmentation can be trained effectively. These results in Table 6 suggest that the proposed method is able to give more target details of the input image for training, it has 3% higher accuracies compared to the method which used random crop and have almost 4% higher accuracies compared to the method which used traditional ways of data augmentation. These results proved that our method is partly corrected.

### 3.6. Third Experiment: Dataset Using Other Models

In the third experiment, we used other models compared with Resnet-50 models in the D3 dataset. The results are summarized in Table 7. 

The results in Table 7 show that with our method, many models can get good performance, and Densenet-121 model have better performance than other popular models, but it may take much time for training. VGG-16 net also performs well. Because of the good performance in Densenet-121, we guess that with deeper networks, further improvement may be attained. 

### 3.7. f1-Score and Misclassified Ships

As shown in Figure 4, we can find that the Bulk Carrier images are bright in its edges and have small length and width. But the Container Ship and Oil Tanker are pertained to be with long length and short width. Therefore, it can be predicted that we could easily classified Bulk Carrier with its size characteristics. However, distinguishing between Container Ships and Oil Tankers may be a challenge.

For further study, we also use precision (the ratio of true positives and predicted positives) and recall (the ratio of true positives and all positives samples) to evaluate our results of D3 dataset, which was trained with Resnet-50 models. And we use f1-score to combine precision and recall into one. The f1-score is then given as

(4) $$f1score=2\times \frac{Precision*Recall}{Precision+Recall},$$

The results of the experiment are summarized in Table 8.

**Figure 14:**
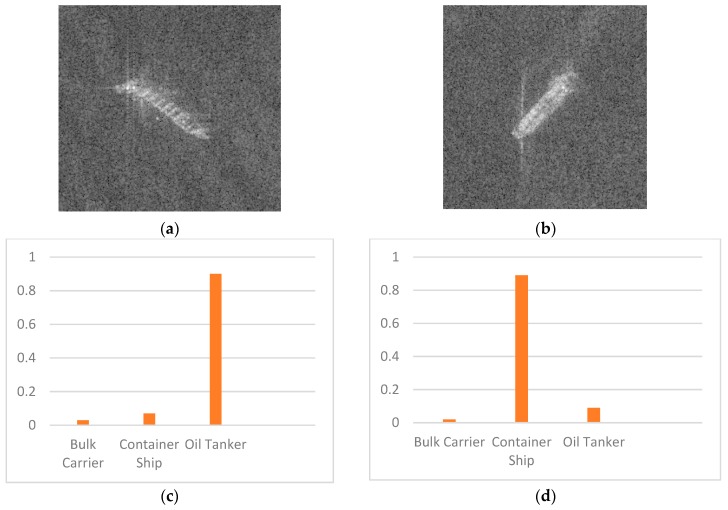
Misclassified ships. (**a**) Container Ship misclassified as Oil Tanker. (**b**) Oil Tanker misclassified as Container Ship. (**c**,**d**) are probabilities of three categories.

Some misclassified samples are shown in Figure 14.

The results in Table 8 show that the classifier has a high f1-score for the classes Bulk Carrier. Due to Bulk Carrier’s distinct shape and size characteristics, it is easy to classify. As shown in Table 8, we can entirely classify the Bulk Carrier images. Consider Container Ships and Oil Tankers, distinguishing between them is a challenge in ship classification, because they have similar shapes and sizes. However, with our method, we can achieve the scores of 0.9555 and 0.9573, which proves that our method performs well in all of the three classes. 

### 3.8. Comparison with Other Methods

The good performance has been shown from the results in Table 2, Table 3, Table 4, Table 5, Table 6, Table 7 and Table 8. To further confirm the importance of combing data processing and training, we did a new experiment. We used the original dataset with no data processing for training and watched the performance with transfer learning and simple CNN models. The results were shown in Table 9.

When compared the results with Table 2, Table 3, Table 4, Table 5, Table 6, Table 7 and Table 8. we can easily find that with no data processing in our dataset, the CNNs cannot perform well either using transfer learning or not. The experimental results confirm that both the data processing and the way in CNN training should be concerned to get better performance.

To confirm our method is effective, we also compare with other methods. In [5], the authors used deep neural networks with transfer learning and fine-tuning for SAR ship classification and they achieved good results. In [17], a multiple input resolution CNN model is proposed and its performance is evaluated. In [38], authors proposed a novel ship classification model combining kernel extreme learning machine and dragonfly algorithm in binary space. The result was shown in Table 9. Because of the dataset and the model, the result may not be exactly correct. When compared our method with these studies, in [5,38], authors did not do data augmentation but training data with fine-tune and a new CNN model. In [17], authors processed data with multiple resolution and used random crop for data augmentation. 

The result in Table 10 shows that our method can get better performance compared with other proposed method by taking a compromise between data processing and CNN training, both in accuracy and f1-score.

### 3.9. Performance in Test Dataset

We also did an experiment with a test dataset. As shown in Table 4, because the test datasets only include 70 images, the data may undulate significantly. The results are summarized in Table 11.

The result shows that with our method, we can also get good performance with a test dataset.

## 4. Conclusions

This paper presented a new method for SAR targets classification on TerraSAR-X high-resolution images. To verify our method, we take several experiments to compare. The experimental results reveal that: (1) compared with the dataset trained via random crop, traditional data augmentation, our method achieve the best performance with regard to classification accuracy. (2) With the proposed method in our dataset, the Densenet-121 model scored the best classification performance with an accuracy of 98.96%, other models like VGG-16, Resnet-50 also perform well. (3) When compared with other researchers’ work, our method can get at least 1% higher accuracies. We also have advantage in f1-score. (4) Our method also has good generalization ability. Our paper has presented the application of our method in ship classification, the point of ship classification is that we cannot get enough high-resolution SAR ship images. Therefore, if we can solve the data problem, we think we can also get good results when promoting our method to other SAR fields with more images and details. Other procedures in the preprocessing step of the images, such as images with GAN, SRCNN, may be the focus of future work.

## Figures and Tables

**Table 1:** Resnet models

Layer Name	Output Size	18-Layer	34-Layer	50-Layer	101-Layer	152-Layer
conv1	112 × 112	7 × 7, 64, stride 2				
conv2_x	56 × 56	3 × 3 max pool, stride 2				
		$\left[\begin{array}{cc} 3\times 3, & 64\\ 3\times 3, & 64 \end{array}\right]$×2	$\left[\begin{array}{cc} 3\times 3, & 64\\ 3\times 3, & 64 \end{array}\right]$×3	$\left[\begin{array}{cc} 1\times 1, & 64\\ 3\times 3, & 64\\ 1\times 1, & 256 \end{array}\right]$×3	$\left[\begin{array}{cc} 1\times 1, & 64\\ 3\times 3, & 64\\ 1\times 1, & 256 \end{array}\right]$×3	$\left[\begin{array}{cc} 1\times 1, & 64\\ 3\times 3, & 64\\ 1\times 1, & 256 \end{array}\right]$×3
conv3_x	28 × 28	$\left[\begin{array}{cc} 3\times 3, & 128\\ 3\times 3, & 128 \end{array}\right]$×2	$\left[\begin{array}{cc} 3\times 3, & 128\\ 3\times 3, & 128 \end{array}\right]$×4	$\left[\begin{array}{cc} 1\times 1, & 128\\ 3\times 3, & 128\\ 1\times 1, & 512 \end{array}\right]$×4	$\left[\begin{array}{cc} 1\times 1, & 128\\ 3\times 3, & 128\\ 1\times 1, & 512 \end{array}\right]$×4	$\left[\begin{array}{cc} 1\times 1, & 128\\ 3\times 3, & 128\\ 1\times 1, & 512 \end{array}\right]$×8
conv4_x	14 × 14	$\left[\begin{array}{cc} 3\times 3, & 256\\ 3\times 3, & 256 \end{array}\right]$×2	$\left[\begin{array}{cc} 3\times 3, & 256\\ 3\times 3, & 256 \end{array}\right]$×6	$\left[\begin{array}{cc} 1\times 1, & 256\\ 3\times 3, & 256\\ 1\times 1, & 1024 \end{array}\right]$×6	$\left[\begin{array}{cc} 1\times 1, & 256\\ 3\times 3, & 256\\ 1\times 1, & 1024 \end{array}\right]$×23	$\left[\begin{array}{cc} 1\times 1, & 256\\ 3\times 3, & 256\\ 1\times 1, & 1024 \end{array}\right]$×36
conv5_x	7 × 7	$\left[\begin{array}{cc} 3\times 3, & 512\\ 3\times 3, & 512 \end{array}\right]$×2	$\left[\begin{array}{cc} 3\times 3, & 512\\ 3\times 3, & 512 \end{array}\right]$×3	$\left[\begin{array}{cc} 1\times 1, & 512\\ 3\times 3, & 512\\ 1\times 1, & 2048 \end{array}\right]$×3	$\left[\begin{array}{cc} 1\times 1, & 512\\ 3\times 3, & 512\\ 1\times 1, & 2048 \end{array}\right]$×3	$\left[\begin{array}{cc} 1\times 1, & 512\\ 3\times 3, & 512\\ 1\times 1, & 2048 \end{array}\right]$×3
	1 × 1	average pool, 1000-d fc, softmax				
FLOPs		$1.8\times 10^{9}$	$3.6\times 10^{9}$	$3.8\times 10^{9}$	$7.6\times 10^{9}$	$11.3\times 10^{9}$

**Table 2:** Traditional augmentation.

Operation	Parameter
Rotate	90,180
Brightening	1.5
Color enhancement	1.5
Contrast	1.5
Sharpness	3.0
Flip	Top bottom

**Table 3:** D1, D2, and D3 dataset.

D1 Dataset			D2 Dataset			D3 Dataset		
Label	Train	Validation	Label	Train	Validation	Label	Train	Validation
Bulk Carrier	896	304	Bulk Carrier	6110	4560	Bulk Carrier	5595	4255
Container Ship	272	128	Container Ship	4080	1920	Container Ship	4114	1936
Oil Tanker	272	128	Oil Tanker	4080	1920	Oil Tanker	4114	1936

**Table 4:** Test dataset.

Label	Test
Bulk Carrier	38
Container Ship	16
Oil Tanker	16

**Table 5:** Datasets using traditional CNN model.

Dataset	Accuracy (%)
D1	91.43
D2	87.49
D3	88.76

**Table 6:** Datasets using Resnet-50 model.

Dataset	Accuracy (%)
D1	94.67
D2	95.43
D3	98.52

**Table 7:** D3 dataset using different models.

Model	Accuracy (%)
Resnet-50	98.52
Alexnet	96.31
VGG-16	98.46
Densenet-121	98.96
Resnet-34	97.24

**Table 8:** D3 dataset with Resnet-50 models.

Label	Precision	Recall	f1-Score
Bulk Carrier	1	1	1
Container Ship	0.9763	0.9355	0.9555
Oil Tanker	0.9381	0.9773	0.9573
Avg. total	0.9715	0.9709	0.9709

**Table 9:** Experiment using original dataset.

Method	Accuracy (%)
Original dataset with simple CNN models	85.71
Original dataset with transfer learning	94.93

**Table 10:** Comparison with other method.

Method	Accuracy (%)	f1-Score
Our Method	98.52	0.9715
Method in [5]	97.62	0.9565
Method in [17]	Unknown	0.9443
Method in [38]	Unknown	0.9404

**Table 11:** Experiment with test dataset.

Accuracy (%)	Classified/Real
98.57	69/70
